# Classifying human emotions in HRI: applying global optimization model to EEG brain signals

**DOI:** 10.3389/fnbot.2023.1191127

**Published:** 2023-10-10

**Authors:** Mariacarla Staffa, Lorenzo D'Errico, Simone Sansalone, Maryam Alimardani

**Affiliations:** ^1^Department of Science and Technology, University of Naples Parthenope, Naples, Italy; ^2^Department of Electrical Engineering and Information Technologies, University of Naples Federico II, Naples, Italy; ^3^Department of Physics, University of Naples Federico II, Naples, Italy; ^4^Department of Cognitive Science and Artificial Intelligence, Tilburg University, Tilburg, Netherlands

**Keywords:** human-robot interaction (HRI), EEG signals, brain-computer interface (BCI), frontal brain asymmetry (FBA), Theory of Mind (ToM), Global Optimization Model (GOM), machine learning, deep learning

## Abstract

Significant efforts have been made in the past decade to humanize both the form and function of social robots to increase their acceptance among humans. To this end, social robots have recently been combined with brain-computer interface (BCI) systems in an attempt to give them an understanding of human mental states, particularly emotions. However, emotion recognition using BCIs poses several challenges, such as subjectivity of emotions, contextual dependency, and a lack of reliable neuro-metrics for real-time processing of emotions. Furthermore, the use of BCI systems introduces its own set of limitations, such as the bias-variance trade-off, dimensionality, and noise in the input data space. In this study, we sought to address some of these challenges by detecting human emotional states from EEG brain activity during human-robot interaction (HRI). EEG signals were collected from 10 participants who interacted with a Pepper robot that demonstrated either a positive or negative personality. Using emotion valence and arousal measures derived from frontal brain asymmetry (FBA), several machine learning models were trained to classify human's mental states in response to the robot personality. To improve classification accuracy, all proposed classifiers were subjected to a Global Optimization Model (GOM) based on feature selection and hyperparameter optimization techniques. The results showed that it is possible to classify a user's emotional responses to the robot's behavior from the EEG signals with an accuracy of up to 92%. The outcome of the current study contributes to the first level of the Theory of Mind (ToM) in Human-Robot Interaction, enabling robots to comprehend users' emotional responses and attribute mental states to them. Our work advances the field of social and assistive robotics by paving the way for the development of more empathetic and responsive HRI in the future.

## 1. Introduction

Several efforts have been undertaken in the last decade to humanize the physical appearance of robotic devices to improve their acceptance among human users. Anthropomorphic robots, in particular, have gained popularity due to their humanlike attributes that facilitate social interaction with humans (Ziemke, 2020). Humanoid robots are often perceived as autonomous agents due to their ability to interact with the environment and display social behavior through verbal and non-verbal channels (Banks, 2020).

However, in order to make robotic devices more relatable to human users, it is necessary to also humanize their behavior. Studies in the field of Socially Assistive Robotics (SAR) have demonstrated that personalizing robot behavior based on the context and user needs would improve its explainability and acceptance by the users (Benedictis et al., 2023). In this regard, the typical approaches for development of humanlike robot behavior are generally inspired by the reproduction of human-human interaction mechanisms (Wallkötter et al., 2021) both at the physical and cognitive levels.

In addition to physical engagement, a special emphasis has been placed in recent years on the cognitive and emotional components of the interaction. It is well-argued in the psychological literature that a person's emotional and/or cognitive states play a significant role in relationships and therefore such mental states must also be considered in social and assistive robotics (SAR) to permit the robot to adapt its behavior accordingly (Iengo et al., 2012). If social robots can comprehend a person's mental state, the field of Human-Robot Interaction (HRI) might reach a new dimension, creating the groundwork for the development of a Theory of Mind (ToM) (Premack and Woodruff, 1978). Equipping robots with this level of cognitive capability may also improve interlocutor engagement in complex social interactions (Staffa and Rossi, 2016).

This skill, either alone or in conjunction with other affective and cognitive capacities, should result in more humanized robotic behavior capable of achieving mutual understanding (MU) between people and robots. To achieve MU, two approaches are necessary; on the one hand, the robots should be able to demonstrate more readable and transparent behavior that would increase interpretability and anticipation by the human users (Wallkötter et al., 2021), and on the other hand, the robots should be equipped with high-level cognitive skills to interpret the human partner's global states (needs, intentions, emotional states) and react to them accordingly. Specifically, the robot's emotional system must be built both in terms of affective elicitation and sensing (Wilson et al., 2016). This can be accomplished by creating robots capable of eliciting emotions through humanized social actions and interpreting human emotions in the same way that a human partner would.

On this topic, a recent survey by Ahmed et al. (2023) exhaustively reviewed the state-of-the-art progress and related works on emotion recognition. The authors indicated that the most used technologies to acquire data from the human user were physiological sensors such as electroencephalogram (EEG), electromyography (EMG), electrodermal activity (EDA), and other smart wearables and mounted emotion recognition tools such as HMDs (Head Mounted Displays). They also discussed different emotion classification methods including machine learning (ML) or deep learning (DL) algorithms for obtaining better recognition accuracy. It was concluded that deep learning models give better detection accuracy than machine learning models. When compared on the same EEG dataset (DEAP; Koelstra et al., 2011), among the machine learning models, SVM was the best machine learning model with the highest detection accuracy of 91.3%, and for deep learning, the best model was the combination of three deep learning algorithms (CNN, RNN, AE) reaching 95% accuracy.

However, it is recently argued that for better detection of users' affective states during HRI, the systems should be trained and validated using neurophysiological data that was collected in HRI settings (Alimardani and Hiraki, 2020). This is in contrast to the majority of existing emotion classification approaches in the literature, which use affective stimuli that users view (e.g., images or videos) and collect data in the absence of a robot. Therefore, research in neurorobotics can investigate humans' neural responses to HRI and establish a deeper understanding of the user's internal states, such as emotions, by employing robots as tools to study and replicate past neuroscientific evidence.

To this end, social robots have recently been integrated with the development of brain-computer interface (BCI) systems to manage a more ecologically valid measurement and analysis of human brain waves specifically for HRI application. While EEG devices have been previously used in the robotics field for robot's motor control (Alimardani et al., 2013; Staffa et al., 2019), very few approaches exist for the classification of a human's internal state during human-robot interaction (Alimardani and Hiraki, 2020; Staffa and Rossi, 2022; Staffa and D'Errico, 2023). For instance, Ogino and Mitsukura (2018) used a machine learning algorithm to identify user emotions from EEG signals when interacting with a robot. Another study Toichoa Eyam et al. (2021) used an EEG-based emotion sensing system to control a robot to perform different actions based on the user's emotions and showed that such an approach can improve human-robot interaction.

In general, studies on the use of EEG in robotic applications have demonstrated the feasibility and potential of this technology with different user groups including children (Alimardani et al., 2021). However, there are still technical challenges to overcome, such as noise reduction in EEG signals, appropriate feature selection, and the complexity of the machine learning algorithms used for emotion recognition. Therefore, this work pursued two goals. First, we aimed to validate neuroscience theories that suggest particular relationships between alpha and beta waves in specific areas of the brain as a metric of a person's emotions during HRI. Particularly, we were interested in evaluating whether a different personality (positive or negative) of the robot could induce a different mental state in the users who interacted with it. This would pave the way for a first attempt to model ToM in HRI using neural activity, allowing the robot to understand users by attributing mental states to them.

Second, we were interested in tackling the commonly faced issues in the classification of EEG signals, such as the bias-variance trade-off, dimensionality, and noise in the input data space. The latter, in particular, may significantly impair the predictive power of the classifiers in real-time and out-of-the-lab settings. While there are several machine or deep learning models that can address some of the issues listed above, combining them could significantly improve the performance of the algorithms. As a result, in this paper, we propose a GOM based on a combination of feature selection and hyperparameter optimization techniques that can improve the overall accuracy of various machine learning and deep learning models on EEG signals.

## 2. Background

### 2.1. Frontal brain asymmetry (FBA)

In Russel's circumplex model of affect (Russell, 1980) emotions are presented in a 2-dimensional valence-arousal space (VAS). Thus, any emotion can be described using an unpleasantness/pleasantness dimension (valence) and a high arousal/low arousal dimension (activation). Related to this, Frontal Brain Asymmetry (FBA), the asymmetry between the left and right brain hemispheres, forms the most prominent expression of emotion in brain signals and has been identified by neuroscience literature as a marker of emotional processing and regulation (Wheeler et al., 1993). FBA suggests that asymmetry in the distribution of neural activity between the brain hemispheres in the frontal region is associated with the valence and arousal of the emotional states (Davidson et al., 1990). By this theory, the frontal area of the brain exhibits differential lateralization and is organized around approach-withdrawal tendencies (the experience of positive affect facilitates approach behaviors and the experience of negative affect triggers withdrawl) (Davidson et al., 1990). In contrast to the right frontal region, which is involved in the experience of negative emotions (lower valence values), such as fear or disgust, the left frontal area is involved in the experience of positive emotions (high values of valence), such as joy or happiness (Davidson et al., 1990; Lewis et al., 2007). Therefore, this metric can be useful in various HRI contexts, such as evaluating the effectiveness of therapeutic interventions with assistive robots (Alimardani et al., 2020).

**Figure 1:**
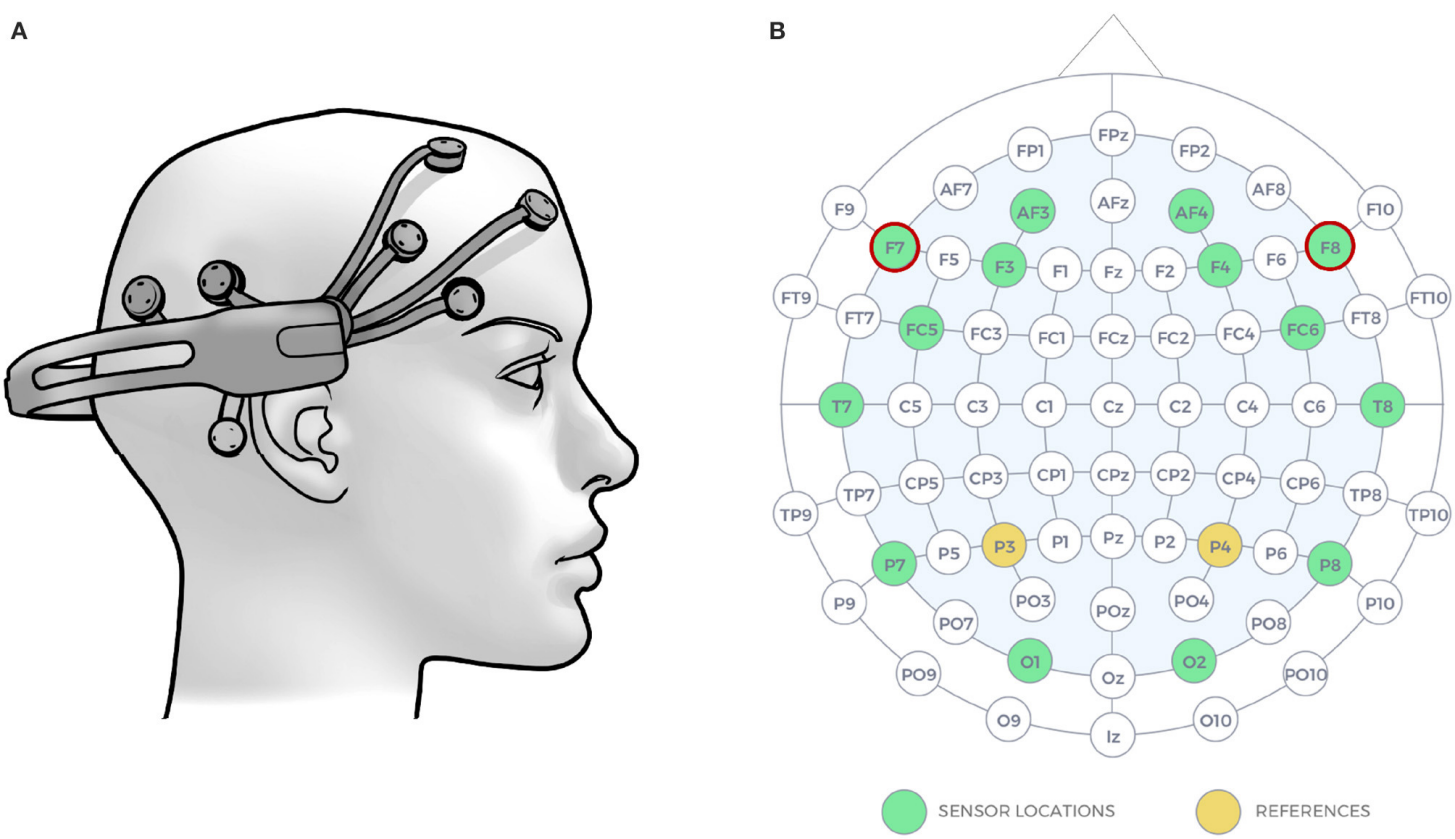
**(A)** Emotiv EPOC+ headset was used for recording of brain activity, **(B)** the EEG signals were collected form 14 electrode sites (shown with green) according to the 10-20 system. The two electrodes with red circles were used for computation of Frontal Brain Asymmetry. The images are taken from the documentation of the Emotiv EPOC+ helmet available at https://www.emotiv.com.

Initially, Davidson et al. (1990) used the asymmetry index *DI* = (*L* − *R*)/(*L* + *R*), where L and R were the power of a specific frequency band in the left and right hemispheres, respectively. Later, the asymmetry concept was extended integrating multiple frequency bands involved in emotional processing: For instance, FBA can be calculated by subtracting the power of EEG signals in the α (8–13 Hz) and β (13–30 Hz) frequency bands extracted from the electrodes placed on the left and right frontal hemispheres (Mühl et al., 2014; Al-Nafjan et al., 2017; Menon et al., 2018; Zhao et al., 2018). The electrodes that are often used to acquire FBA are typically F3, F7, or AF7 from the left hemisphere and F4, F8, or AF8 from the right hemisphere according to the International 10-20 EEG positioning system (see Figure 1). Below, we show four equations adapted from Al-Nafjan et al. (2017) that are used for computation of emotional valence (Equations 1, 3, 5, 7) and arousal (Equations 2, 4, 6, 8) from EEG signals. Here, *α*_*F*7_, *α*_*F*8_, *β*_*F*7_, and *β*_*F*8_ indicate the alpha and beta band powers measured from F7 and F8 channels.


(1) $$\begin{array}{l} v_{1}=\frac{\alpha_{F8}}{\beta_{F8}}-\frac{\alpha_{F7}}{\beta_{F7}} \end{array}$$

(2) $$\begin{array}{l} a_{1}=\frac{\alpha_{F7}+\alpha_{F8}}{\beta_{F7}+\beta_{F8}} \end{array}$$

(3) $$\begin{array}{l} v_{2}=ln\left(\alpha_{F7}\right)-ln\left(\alpha_{F8}\right) \end{array}$$

(4) $$\begin{array}{l} a_{2}=-\left(ln\left(\alpha_{F7}\right)+ln\left(\alpha_{F8}\right)\right) \end{array}$$

(5) $$\begin{array}{l} v_{3}=\frac{\beta_{F7}}{\alpha_{F7}}-\frac{\beta_{F8}}{\alpha_{F8}} \end{array}$$

(6) $$\begin{array}{l} a_{3}=log_{2}\left(\frac{\beta_{F7}+\beta_{F8}}{\alpha_{F7}+\alpha_{F8}}\right) \end{array}$$

(7) $$\begin{array}{l} v_{4}=\alpha_{F8}-\alpha_{F7} \end{array}$$

(8) $$\begin{array}{l} a_{4}=\frac{\beta_{F7}+\beta_{F8}}{\alpha_{F7}+\alpha_{F8}} \end{array}$$

### 2.2. Emotion classifiers

Over the last decade of research on emotion recognition using physiological signals, researchers have deployed numerous methods of classifiers to assess the different types of emotional states, stress levels, and engagement levels. In a review of EEG-based emotion recognition approaches (Suhaimi et al., 2020), the authors point out that Support Vector Machine (SVM) and K-Nearest Neighbor (KNN) are among the most popular methods for emotion classification, with the highest achieved performance being 97.33% (SVM) and 98.37% (KNN). However, there were also other algorithms used for emotion classification that performed equally well, such as Random Forests (RF) (98.20%), Dynamic Graph Convolutional Neural Network (DGCNN) (90.40%), Fisherface (91.00%), and Long-Short Term Memory (LSTM) (92.23%).

The aforementioned findings highlight the best models for emotion recognition tasks, but they do not accurately reflect the overall agreement between task-specific EEG features because some algorithms performed best in the generalized arousal and/or valence dimensions, while others relied on highly specific emotional markers. Comparing the actual classification effectiveness of all the various classifiers is so difficult. We decide to optimize and compare various classifiers for the same task using a global optimization technique, concentrating on the top-rated algorithms. The classifiers selected in this study were: SVM with four different kernels (Linear, Polynomial, RBF, and Sigmoid), KNN, Decision Tree (DT), RF, and Multi-Layer perceptrons (MLP). These models have previously shown excellent results in stress and emotion classification from EEG signals (Khosrowabadi et al., 2011; Wu et al., 2017; Al shargie et al., 2018; Nishtha et al., 2022; Rajendran et al., 2022).

### 2.3. Models optimization

In machine learning, hyper-parameter optimization (Feurer and Hutter, 2019) is the process of choosing the best values for parameters that control the learning process, which can vary depending on the type of machine learning model used. The goal is to find the optimal combination of hyper-parameter that minimize a loss function on independent data (Claesen and De Moor, 2015), often estimated using cross-validation (Bergstra and Bengio, 2012). There are two common basic methods for hyper-parameter optimization (Bergstra et al., 2011):

- *Grid Search*: the traditional hyper-parameter optimization is the grid search, which involves exhaustively searching a manually specified subset of a learning algorithm's hyper-parameters. Performance is evaluated through cross-validation or evaluation on a validation set. If necessary, limits and discretization must be set manually prior to grid search;
- *Random Search*: random search replaces exhaustive enumeration with random selection of combinations. It can be applied to discrete, continuous and mixed spaces, and can outperform grid search in cases where only a limited number of hyper-parameters impact the final performance of the machine learning algorithm. The optimization problem is said to have low intrinsic dimensionality in such cases. Random search is easily parallelizable and allows for incorporation of prior knowledge by specifying the sampling distribution. Despite its simplicity, random search remains a significant baseline method for comparing performance of new hyper-parameter optimization methods.

In this work, we used the grid search method for optimizing SVM, DT, KNN and RF models while, due to its huge dimension in the research space, for the MLP model a kind of random search known as *hyperband* from Keras has been chosen. The implementation of the methods mentioned above has been performed using the Python library Scikit-learn which offers two different generic algorithms for this purpose: *GridSearchCV*, for exhaustive parameters combination and *RandomSearchCV*, for sampling a fewer number of parameters in a parametric space with a specified distribution. Considering the computational resources available and the accuracy of the method, the *GridSearchCV* algorithm has been selected. Note that it integrates within it a cross-validation process known as *LeaveOneGroupOut*.

## 3. Experimental procedure

### 3.1. Participants

A sample of 10 Italian native-speaker (7 males and 3 females with ages ranging from 20 to 42) participated in the experiment. Upon arrival, participants signed an informed consent in which the complete procedure was detailed.

### 3.2. EEG recording

EEG signals were gathered by the Emotiv EPOC+ headset, which is a non-invasive 14-channels (AF3, AF4, F3, F4, FC5, FC6, F7, F8, T7, T8, P7, P8, O1, O2) EEG device characterized by lightweight and wireless connection with a sampling rate of 128 Hz. In this study, we use all the 14 channels of the headset, but only channels F7 and F8 were used for computing valence and arousal metrics as shown by red circles in Figure 1.

### 3.3. Conditions

In the proposed study, we used a humanoid robot Pepper endowed with two opposite personalities (positive and negative) to induce different levels of emotional states in participants. All participants experienced both conditions in a within-subjects study design.

Due to the constraints of the Pepper robot, which does not allow facial expressions, we implemented the robot's personalities by modeling voice, dialogues, head, and bodily movements (see Table 1). Specifically, we used brief non-linguistic vocalizations, such as laughter and short intake of surprise breath for positive personality, and negative “oh” and repeated long intakes of sudden breath for negative personality. The advantage to use non-linguistic utterances is that they are universally associable with different emotional meanings regardless of language and culture (Read and Belpaeme, 2012), thus can be used in different experimental settings without the need to customize the robot voice characteristics.

**Table 1:** Design of the robot personalities using vocalization and bodily movements.

		**Robot behavior**		
		**Voice and dialogue**	**Head and eyes**	**Body**
Robot personality	Positive	Laughter vocalization and short intake of surprise breath	Straight head with green-colored eyes	Upright posture, vertical and lateral extension with large and fast movements
	Negative	Negative “oh” and long intake of suddenbreath	Forward head bend with red-colored eyes	Forwards-leaning posture, arms at side of trunk, less energetic and smaller gestures

The dialogue was set up as a simple conversation that one can have in the morning with a colleague or a friend, characterized by an initial greeting and questions on how one feels, the plans for the day, etc. The dialogue of the positive robot was enriched with some forms of humor to elicit positive and engaging reactions (Niculescu et al., 2013), while the negative robot's dialogue was characterized by fewer interactions, no humor, and longer pauses.

The head and bodily movements of the robot were designed following Coulson's (Coulson, 2004) and Kleinsmith's (Kleinsmith and Bianchi-Berthouze, 2013) works where it was shown that specific features such as postures or gestures and their relative dynamics (e.g., speed and amplitude) could communicate particular emotions. Therefore, an upright posture with large and fast movements as well as green-colored eyes was implemented to communicate a positive personality of the robot, whereas, for a negative personality, the robot presented a forward-leaning posture, red-colored eyes, and less energetic and smaller gestures.

### 3.4. Procedure

**Figure 2:**
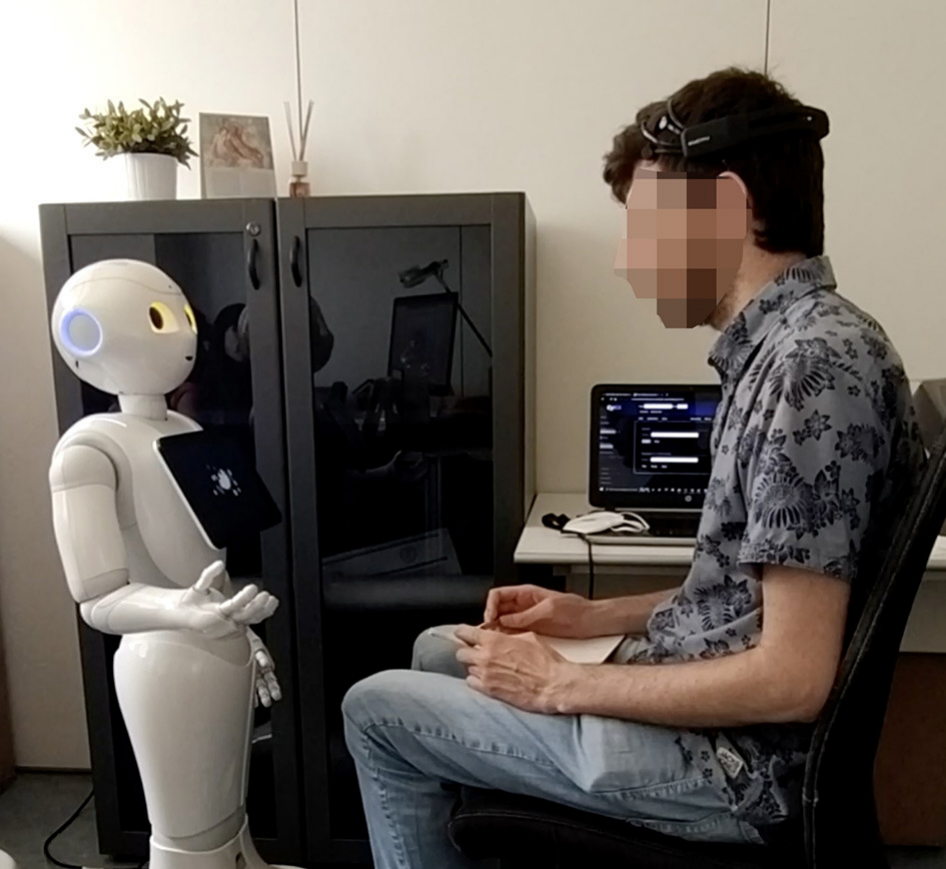
An overview of the experimental setup: the participant was seated in front of a Pepper robot, while wearing the Emotiv EPOC+ headset for EEG recording.

Following instructions and the acquisition of consent, the experimenter placed the EEG headset on the participants' heads and checked the signal quality. Participants were then seated in front of a Pepper robot (Figure 2) and interacted with it under two experimental conditions that entailed two different robot personalities. The robot showed different arm gestures, voice, and posture according to Table 2 to induce either a positive or negative emotional state in the human partner. The order of conditions was randomized and each scenario lasted about 10 min. The timing of the administration was regulated by internal timers that were set empirically. Therefore, if the participant did not interact after a while, the session continued when those internal timers expired.

**Table 2:** Best classification performances obtained by the Global Optimization model. Percentile refers to the feature subset and pred_time indicates the model's response time.

**Classifier**	**Percentile**	**Accuracy**	**Precision**	**Recall**	**F-score**	**pred_time(s)**
SVM_linear	5	0.92	0.93	0.92	0.92	2.00E-05
SVM_poly	5	0.92	0.92	0.92	0.91	1.50E-05
SVM_RBF	5	0.92	0.92	0.92	0.91	4.30E-05
SVM_sigmoid	5	0.92	0.92	0.92	0.91	1.36E-07
MLP	5	0.91	0.92	0.91	0.91	2.92E-05
KNN	50	0.91	0.91	0.91	0.89	1.76E-04
RF	5	0.89	0.91	0.89	0.89	3.80E-06
DT	15	0.84	0.86	0.84	0.86	7.20E-08

The EEG signals collected in each HRI scenario were labeled according to the robot's personality; label 0 for data from the interaction with the negative robot and label 1 for data collected during the interaction with the positive robot. EEG Features were then extracted from the signals and used to train the emotion classification models in a supervised learning manner.

## 4. Data analysis

The data collected by experimental sessions is composed of 2,048 records (balanced) for each of the 10 experimental subjects, yielding a total of 20,480 entries. The data, in its original form, consists of vectors of 148 floating point features and is classified according to two possible classes (positive, negative).

### 4.1. Signal pre-processing

The training of a BCI system is normally preceded by a phase of signal pre-processing where noise and movement artifacts are removed from the signals before a feature vector corresponding to the classification problem is constructed. To remove the noise, we applied a Butterworth bandpass filter with cutoff frequencies at 0.16 and 43Hz, which is the recommended bandwidth range for the Emotiv EPOC+ headset (Badcock et al., 2013). Additionally, we applied an IIR filter to remove the DC Offset in the signals.

### 4.2. Feature extraction

Feature extraction was performed on all 14 channels recorded by the EEG headset. First, a band-pass filter was applied to the signals to decompose them into the following 5 frequency bands: σ (0.54Hz), θ (48Hz), α (814Hz), β (1430Hz), and γ (3040Hz). This resulted in 70 decomposed signals per window, 5 for each of the 14 channels of the headset. Next, a periodigram function was applied to the decomposed signals to calculate the power spectral density (PSD). Therefore, each signal was converted into the relative power distribution on the frequency axis, from which statistical information such as mean and standard deviation were extracted. This procedure was repeated for all five frequency bands, yielding a total of 140 features. All the channels were used to extract frequency band features. On these frequency bands mean and standard deviation of the PSD have been computed obtaining a total amount of 140 features plus the 8 features obtained by computing valence and arousal on alpha and beta frequency bands Finally, the 8 features described in Section 2 (v1 to v4 for valence characteristics and a1 to a4 for arousal characteristics) were extracted and added to the feature vector. To summarize, each EEG record was transformed into a feature vector including 148 features.

### 4.3. Feature normalization

EEG spectral features can have a wide range of values due to individual differences, channel locations, and frequency ranges. Therefore, feature normalization can help to reduce the impact of outliers and make the data more suitable for the ML algorithms. Min-max normalization is a common technique used to scale data to a specific range, often between 0 and 1 or between −1 and 1. This technique is useful to maintain the original scale of the data that is uniformly distributed. The normalization of the features in the range of [0, 1] was performed by applying the following formula to each of the 148 features.


(9) $$\begin{array}{l} x^{\prime }=\frac{x-min\left(x\right)}{max\left(x\right)-min\left(x\right)} \end{array}$$

This normalization process inevitably involves information loss due to the difference in power values measured among different channels and frequency bands. This loss of information is partially recovered by the eight valence and arousal features, which describe the asymmetry between the α and β band powers in the right and left frontal hemispheres.

Following visualization of the normalized features, it was observed that excessively high values of some features flattened the distribution of normalized data toward zero. To reduce the impact of such outlier features, the following rule was applied: the value of 5% of the highest measurements was replaced by the maximum value of the remaining 95% of the measurements for each feature.

## 5. Global Optimization Model (GOM)

To improve emotion classification performance from EEG data, we adopted a GOM which is a classification approach characterized by a process that combines feature selection and hyperparameter optimization techniques (Boubezoul and Paris, 2012; Ren et al., 2016). We considered 8 models for classification: Support Vector Machines (SVM) with 4 different kernels (i.e., *linear, poly, RBF, sigmoid*), Decision Tree (DT), K-Nearest Neighbors (KNN), Random Forests (RF), and Multi-Layer Perceptrons (MLP). Below, the grid of possible values for each hyperparameter of each model is reported:

- **SVM-linear**: ('kernel': ['linear']; 'C': [0.1, 1, 10]; 'tol': [1e-4, 1e-3, 1e-2]; 'shrinking': [True, False])
- **SVM-poly**: ('kernel': ['poly']; 'C': [0.1, 1, 10]; 'degree': [2,3,4]; 'gamma': ['scale', 'auto']; 'tol': [1e-4, 1e-3, 1e-2]; 'shrinking': [True, False])
- **SVM-rbf**: ('kernel': ['rbf']; 'C': [0.1, 1, 10]; 'gamma': ['scale', 'auto']; 'tol': [1e-4, 1e-3, 1e-2]; 'shrinking': [True, False])
- **SVM-sigmoid**: ('kernel': ['sigmoid']; 'penalty': ['11', '12']; 'loss': ['hinge', 'squared_hinge']; 'tol': [1e-4, 1e-3, 1e-2]; 'max_iter': [1000, 2000])
- **DT**: ('criterion': ['entropy']; 'splitter': ['best', 'random']; 'max_depth': [3, 6, 9, 12, 15, None]; 'min_samples_split': [2, 3, 4, 5]; 'min_samples_leaf': [1, 2, 3, 4]; 'max_features': ['auto', 'sqrt', 'log2', None]; 'ccp_alpha': [0.0, 0.010, 0.020, 0.030])
- **RF**: ('criterion': ['entropy']; 'max_depth': [3, None]; 'min_samples_split': [2]; 'min_samples_leaf': [1]; 'n_estimators': [50, 100, 200]; 'bootstrap': [True, False]; 'oob_score': [True, False]; 'warm_start': [True, False]; 'ccp_alpha': [0.0])
- **KNN**: ('n_neighbors': [5, 10, 20]; 'weights': ['uniform', 'distance']; 'algorithm': ['auto', 'ball_tree', 'kd_tree', 'brute']; 'leaf_size': [30, 60, 120]; 'p': [1,2,3])
- **MLP**: ('n_layers': [1, 2, 4, 5]; 'n_dense_values': ['min'=32, 'max'=1024, 'step'=8]; 'drop_out_values': ['min'=0, 'max'=0.9, 'step'=0.1]; 'optimizer': ['adam', 'adamax']; 'output_activation': ['softmax', 'sigmoid']).

### 5.1. Feature selection

Univariate feature selection (i.e., the selection of the best features based on univariate statistical tests) was used as a technique to perform feature selection for global optimization. The *SelectPercentile* function from Scikit-learn library was chosen to implement this method. This function selects a user-specified percentage of features that reach a high *F*-value in ANOVA tests. In this work, feature selection was executed as a function of the percentile variable in the range [5, 100] with steps of 5.

### 5.2. Hyperparameter optimization

Models were validated using leave-one-subject-out cross-validation (LOOCV), in which the model is trained on all but one subject and tested on the subject left out. This process is repeated for every subject in the dataset. LOOCV is a special form of CV where the value of k is equal to the number of samples in the dataset. This process is repeated for each proposed classification model to select the best subset of features an optimal combination of hyperparameters. As a result, 1600 cross-validations (10 subjects × 8 models × 20 percentiles) were performed to determine which of the optimized models, differentiated by type and subset of features, was globally the best at solving the emotion classification problem at hand.

The global optimization algorithm (see Algorithm 1) iterates on each of the basic models, while optimizing the model performance by obtaining a subset of the best features and tuning the model hyperparameters to that feature subset. Subsequently, models are evaluated by cross-validation, splitting each dataset into subsets relating to different experimental subjects. This process is repeated for each classification model proposed so that the best subset of features, and optimal combination of hyperparameters are chosen for different types of models.

**Algorithm 1:**
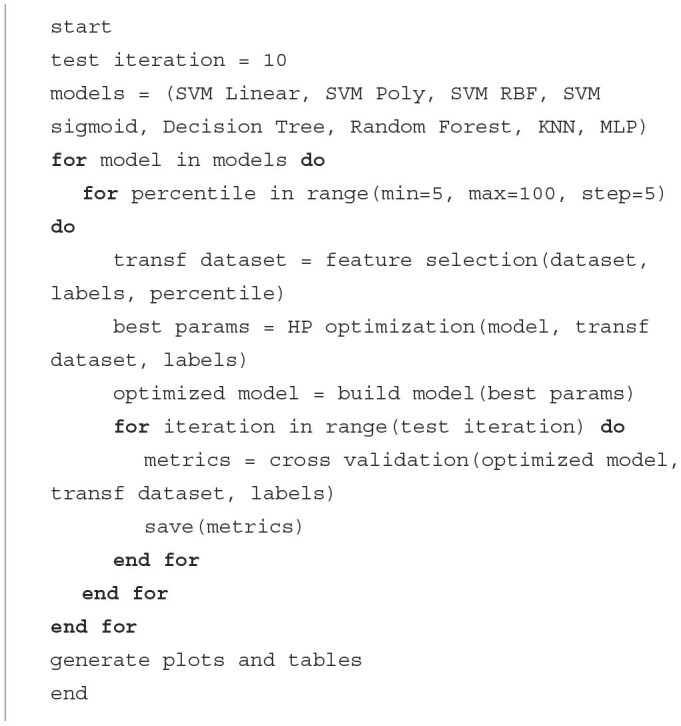
Pseudo-code.

## 6. Results

To evaluate the performance of the proposed approach, we computed the average metrics of accuracy, precision, recall, and F-score from all models. Additionally, the prediction time is reported to indicate the computational cost of the classification task for each optimized model.

### 6.1. Model performance

Table 2 provides a summary of the highest performance obtained by each of the 8 classifiers after optimization. The first column shows the percentile of the feature vector with which the optimized model was trained and tested, whereas the last column shows the average response time of the model in the classification task for a single input. All SVM models could achieve the highest accuracy of 92% on the positive vs. negative emotion classification task regardless of their kernels. however the *SVM_sigmoid* model was about 100 to 200 times faster and so it can be considered the best model in terms of accuracy/time ratio. While *random_forest* and *decision_tree* also were fast and required fewer computing resources, they yielded poor accuracy compared to the rest of the models.

### 6.2. Selected features

**Figure 3:**
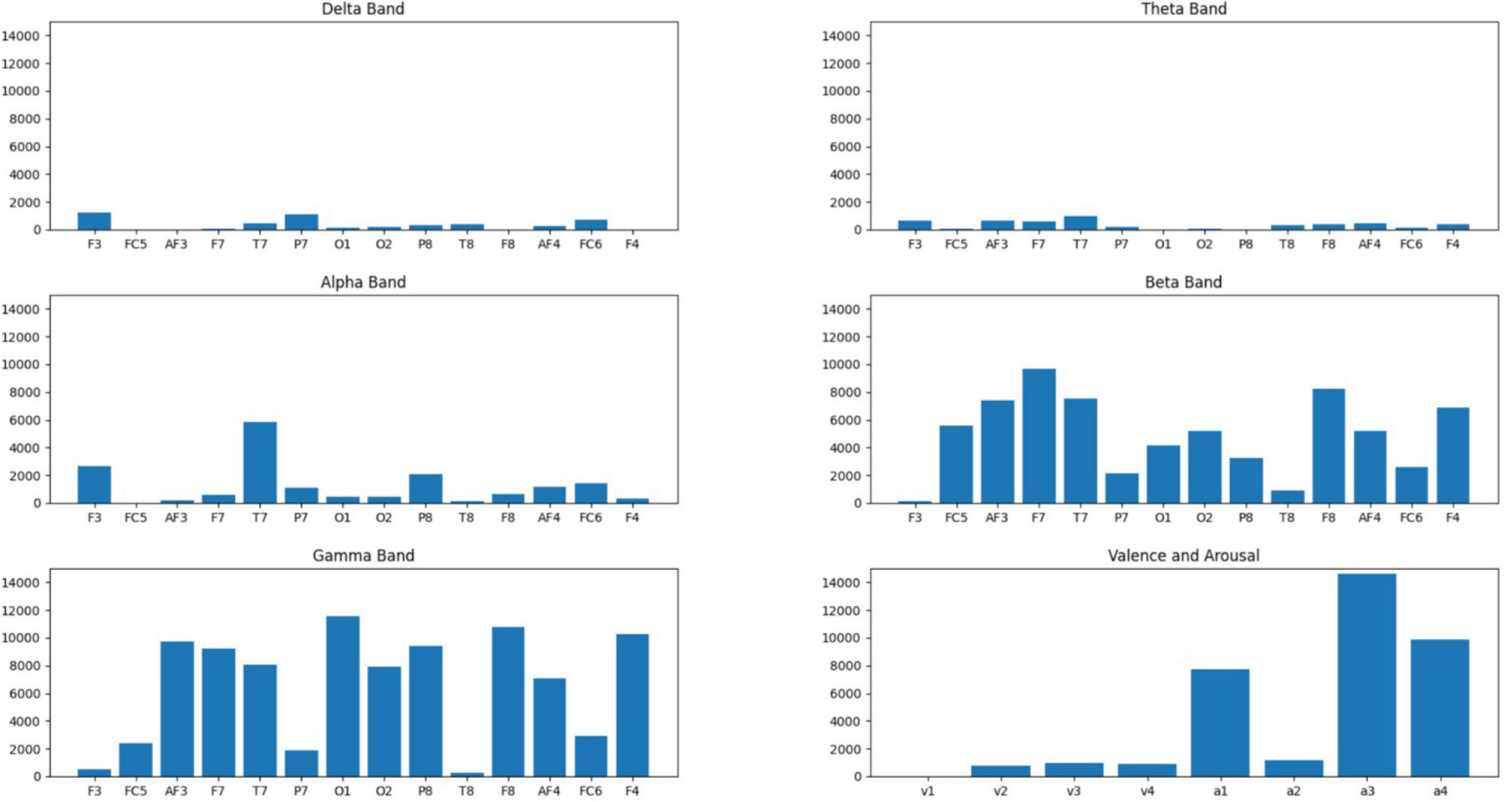
Score results of F-ANOVA test (Y-axis) performed for each frequency band (alpha, beta, gamma, delta, theta) for each of the 14 channels recorded plus valence-arousal values (X-axis).

Figure 3 presents the scores computed by the ANOVA tests for each feature. Surprisingly, compared to the literature in which alpha and beta frequency bands are reported as the most predictive, gamma waves obtain the highest F-scores among the frequency bands, along with beta and then alpha waves. Additionally, confirming what was discussed regarding FBA (concerning the task reported in this study), arousal values are the highest and therefore the most predictive for classification purposes.

### 6.3. Neuromarkers of emotion

**Figure 4:**
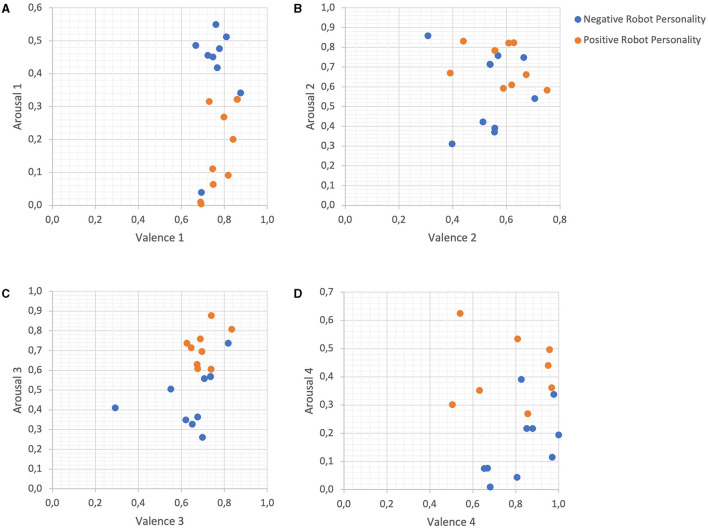
Distribution of arousal and valence features per emotional state. Scatterplots are made using pairs of arousal and valence values obtained from Equations **(A)** (1–2), **(B)** (3–4), **(C)** (5–6), **(D)** (7–8), respectively.

Figure 4 presents four scatter plots showing the mapping of the classified emotional states (positive vs. negative) on the two-dimensional arousal-valence feature space. Each subpanel in this figure is composed of arousal and valence features that were obtained by Equations (1)–(8). Blue circles indicate the feature values during interaction with the robot with a negative personality, whereas red circles are representative of the feature values detected during interaction with the positive robot. As can be seen in this figure, almost all variations of the FBA metrics adapted from Al-Nafjan et al. (2017) yielded quite separable clusters of responses for negative and position emotional states. This inter-class separability of data points within the arousal-valence feature space enables efficient localization of the decision hyperplane and hence can explain the high prediction accuracy by SVM models on the emotion classification task at hand.

## 7. Discussions and conclusions

The main goal of this work was to estimate users' emotional states from EEG brain activity when they interacted with a robot with positive or negative personalities. In particular, by extracting Frontal Brain Asymmetry (FBA) as neuromarkers of emotional valence and arousal and employing a global optimization model for feature extraction, we aimed to develop a reliable emotion classifier for HRI interactions. Our results demonstrated a generally high performance (92% accuracy) obtained by the optimized models with only a small subset of the feature space (5 percentile) that included FBA neuromarkers.

The results from this study validate our hypothesis regarding the possibility of EEG-based emotion recognition in HRI scenarios. Classification of emotional states using brain activity has been widely studied in affective computing and brain-computer interfacing literature (Al-Nafjan et al., 2017; Torres et al., 2020; Rahman et al., 2021; Wang and Wang, 2021), however very little is known about the potentials and challenges associated with the application of such neurotechnology in HRI settings (Alimardani and Hiraki, 2020). Traditionally, HRI research relies on behavioral modalities such as facial expressions, bodily gestures, and voice to infer a human user's emotions during interaction with social robots (Spezialetti et al., 2020). These techniques are often computationally expensive, require privacy-intrusive sensors such as cameras and microphones in the room, and may not give an accurate evaluation of core emotional states that are not behaviorally manifested. Our results indicate that even with a commercial EEG headset such as Emotiv EPOC+, it is possible to classify users' emotional responses to a robot's negative and positive behavior with a relatively high accuracy. This provides an opportunity for future research to integrate fairly inexpensive and easy-to-use EEG headsets as a new tool for offline evaluation of the interaction as well as real-time monitoring of the user's mental state. By employing such real-time BCI systems in HRI, it is then possible to endow robots with appropriate social skills and adaptive behavior that would maximize the efficiency and quality of interaction (Prinsen et al., 2022).

To classify the emotional responses of users from EEG signals, we proposed a global optimization model based on a combination of feature selection and hyperparameter optimization techniques applied to various classification methods, including not only classical machine learning models such as Support Vector Machines and Decision Trees but also deep learning models such as the Multi-Layer Perceptron. The results validated neuroscience theories about the involvement of the FBA including the alpha and beta brain waves in the detection of emotional responses (Al-Nafjan et al., 2017; Alimardani et al., 2020; Kuijt and Alimardani, 2020; Wang and Wang, 2021). This was further supported by Figure 4, where FBA-based neuromarkers of arousal and valence demonstrated a clear separation of the user's negative and positive mental states. Moreover, a significant contribution of the gamma band activity was observed (Figure 3), which corroborates past research indicating the involvement of this frequency band in emotional processing (Hu et al., 2020).

One explanation for the high accuracy achieved by the classification models employed in this study, particularly SVMs, could be the binary nature of the classification task (i.e., positive vs. negative). In affective computing literature, emotion classification tasks are often defined as the prediction of discrete emotion labels (e.g., joy, anger, sadness, fear, etc.) (Liu et al., 2017) or continuous mapping of affect within the arousal-valence bi-dimensional space (Nicolaou et al., 2011; Garg and Verma, 2020), whereas, in this study, we simplified the classification problem to a binary prediction with two valence classes; positive vs. negative. While a simple binary classification might be sufficient for application domains that do not require extensive awareness of the user's affective states, this approach bears the specificity limitation in that it does not give an insight into the nuances of the emotional state, for instance, the difference between a sad or angry experience induced by the robot. Additionally, this approach does not allow for capturing the individual differences in emotional experiences. Particularly that the sample of the current study was limited in size (only 10 people) and hence the obtained results can only be discussed in the context of subject-dependent classification across the recruited sample.

### 7.1. Limitations and future perspectives

While the proposed method in this study yielded promising results in the estimate of users' emotional states from EEG brain activity during HRI, it comes with several limitations that should be considered.

The first limitation is the choice of the EEG hardware employed in this study. The flexible structure of the Emotiv Epoc+ EEG headset can lead to movement on the subject's head during experiments, introducing noise during data collection. The headset has a limited number of electrodes (i.e., 14 channels) which gives a low spatial resolution and requires precise electrode placement for accurate readings. The electrodes' short hydration duration affects signal quality throughout the experiment, contributing further noise. Moreover, electrode oxidation due to manufacturing material can lower conductive capacities and signal cleanliness, leading to potential interruptions in data acquisition.

Additionally, subject-dependent factors pose challenges in EEG and HRI studies, as some subjects may respond randomly or put forth minimal effort during the task, making it difficult to determine their actual involvement in the task and subsequently their affective responses. Individuals' physiological responses to the task can vary, limiting the model's adaptability to diverse subjects. Moreover, external factors such as stress or anxiety can overlap with targeted emotional states, potentially impacting the results.

To address these limitations, certain improvements can be implemented in future research. Firstly, using higher-quality instrumentation, such as research-fidelity EEG devices with more electrodes and stable fastenings, would result in more accurate and consistent measurements. With the advancement of neurotechnology, new EEG hardware with dry electrodes has been introduced and validated for research (Mullen et al., 2015; Pontifex and Coffman, 2023) that can be alternatively used in HRI experiments. Additionally, employing electrodes with extended hydration capabilities (such as those requiring conductivity gel) would improve data quality.

To enhance the classification capacity of predictive models, a larger and more diverse sample size of experimental subjects (e.g., 100 or more) should be considered. Future research should attempt the collection of larger EEG datasets from various affective HRI settings that would enable the validation of neuromarkers that could robustly differentiate emotions across users and contexts. Such neuromarkers can then be employed in subject-independent classification models for plug-and-play integration of BCIs in real-time human-robot interaction. In addition to EEG, future research could benefit from the integration of other (neuro)physiological sensors (e.g., heart rate, galvanic skin response, respiration, etc.) for multi-modal classification of emotions (Zhang et al., 2020).

An interesting direction for future research could be focused on the use of various tasks and emotion induction techniques via robot behavior design in order to verify the validity of the measured affective responses toward the robot. Consequently, customizing tasks based on each subject's interests and abilities would promote active engagement and improve the overall quality of the collected data. Furthermore, conducting a preliminary assessment of the subject's basic emotional state through a specific self-completed questionnaire would aid in excluding individuals with strong perennial emotional states that could significantly influence the data.

In conclusion, the current study demonstrated a successful implementation of a global optimization model for the classification of a user's emotional states from EEG brain activity during positive and negative interactions with a social robot. By addressing the aforementioned limitations and implementing the suggested improvements, the proposed method can achieve higher accuracy and reliability in various HRI scenarios. Future development of this work consists of carrying out closed-loop experiments involving adaptive behavior and/or implicit feedback from the robot based on the user's affective states. Additionally, by designing various interaction tasks, we intend to collect EEG data in a broader affective context and probe the impact of the robot's feedback on the user experience and task performance.

## Data availability statement

The raw data supporting the conclusions of this article will be made available by the authors, without undue reservation.

## Ethics statement

Ethical approval was not required for the studies involving humans because (1) The study is not a clinical study, but rather research involving the collection of anonymous data. (2) The data is anonymized. (3) Personally identifiable data is not used nor published. (4) Participants involved in the study, being University students, are above the legal age of consent (aged over 18). (5) Participation in the study was voluntary; participants were free to opt in or out of the study at any point in time. (6) Participants signed an informed consent form specifying the purpose behind the study before they agreed or declined to join. (7) Concerning the image presented in the manuscript, it was pixelated to protect the privacy of the participant in such a way that s/he could not be identified. (8) There was no potential risk of physical, social, or psychological types of harm. The studies were conducted in accordance with the local legislation and institutional requirements. The participants provided their written informed consent to participate in this study.

## Author contributions

MS conceived of the presented idea, provided substantial contributions to the conception and design of the work, wrote the first draft of the manuscript, and supervised the project. MS, LD'E, and SS contributed to the design and implementation of the research, to the analysis of the results, and to the writing of the manuscript. SS carried out the experiments. MA revised it critically for important intellectual content. MS, LD'E, SS, and MA wrote sections of the manuscript. All authors contributed to manuscript revision, read, and approved the submitted version.
