# Supplementary material for: Classifying human emotions in HRI: applying global optimization model to EEG brain signals
Source: Front Neurorobot. 2023 Oct 10;17:1191127. doi: 10.3389/fnbot.2023.1191127 (PMC10595007; doi:10.3389/fnbot.2023.1191127)
Supplement: Supplementary file 1 [file Data_Sheet_1.pdf]

Supplementary Material

1 SIGNAL PROCESSING

1.1 DC Offset suppression: the Butterwoth Filter

Like any wave, brain waves are subject to different types of noise: the best known is the so-called Direct Current Offset (DC Offset). It indicates a shift of the signal from zero in terms of average amplitude: if it's zero, then there's no DC noise. During brain activity, this type of noise can occur in response to even minor changes in the potential of the human body. Data recorded by the helmet measures a DC level of about 4180 units, which, in the internal ADC, is measured through the LSB (Least Significant Bit): 1 LSB is worth about $0.51\,\mu\mathrm{V}$ differentiating signal from noise. However, the absolute value of the offset could also be higher due to the DC Drift effect. In this work, the noise removal procedure is divided into two fundamental phases: (i) in the first phase, a Butterworth filter Butterworth et al. (1930), in the high-pass version with a cutoff frequency of $0.16\,\mathrm{Hz}$ was used, while (ii) in the second phase, an IIR filter was applied to remove the DC Offset. The Emotiv SDK minimally removes DC noise through a high-pass filter, but, as recommended by Emotiv itself, it is advantageous to apply other algorithms to clean up the signal as much as possible. One possibility could be to subtract the average value from the entire channel, but the necessary accuracy is not guaranteed since it could increase the low-frequency noise. The high-pass filter at $0.16\,\mathrm{Hz}$, on the other hand, allows both to remove the background noise and cancel any long-term drift, unlike the average method. There are many other types of noise, from environmental to physiological. Among the environmental ones is the interference caused by electric generators, which varies from country to country. The filter used by the SDK is a Double Notch Filter at 50-60 Hz that affects frequencies below $45\,\mathrm{Hz}$, so using $43\,\mathrm{Hz}$ as the upper limit ensures a perfectly flat spectral response. Furthermore, to eliminate potential interference from the harmonics of electrical components, frequencies above $85\,\mathrm{Hz}$ are also filtered. The Butterworth filter, also called Maximally Flat Magnitude Filter, is a signal processing filter widely known for being able to obtain on one hand a frequency response as flat as possible in the range of frequencies covered by the filter (pass-band) and on the other hand a convergence to 0 in the stop-band. Butterworth showed that a low pass filter could be structured so that the cutoff frequency was normalized to one radian per second and the frequency response was equal to:

$$G(\omega) = \frac{1}{\sqrt{1+\omega^{2n}}} \tag{S1}$$

where ω is the angular frequency in radians over a second and n is the order of the filter Butterworth et al. (1930). The formula can be rewritten considering the cutoff angular frequency ω_c as:

$$|H(j\omega)| = \frac{1}{\sqrt{1+(\frac{\omega}{\omega_c})^{2n}}} \tag{S2}$$

where $H(j\omega)$ is the transfer function which has as input the angular frequency ω equal to $2\pi f$ (with f the frequency for which the filter is calculated). The angular cutoff frequency ω_c, on the other hand, is equal to $2\pi f_0$, with the cutoff frequency f_0. This formulation, which expresses a low-pass filter, can be modified to obtain high-pass, band-pass and band-stop filters Butterworth et al. (1930). In particular, by replacing the capacitances with inductances, the high-pass version is obtained. In this work, the application of the Butterworth filter is performed by using two functions:

1. Python 2.7 scipy.signal library offers the Butter function, defined as:

```
scipy.signal.butter(N, Wn, btype='low',
analog=False, output='ba')
```

in which the parameters have been tuned to obtain a high-pass filter, through the parameter *btype = 'highpass'* with a cutoff frequency ω_n equal to 0.16. The function calculates the numerator and denominator polynomials of the filter, respectively identified by b and a. Then the *lfilter* function was used, defined as:

```
scipy.signal.lfilter(b, a, x, axis=-1,
zi=None)
```

which takes the parameters b and a calculated by the previous function and examines the data along a dimension with an IIR digital filter [1]. The function is implemented internally as a direct transposed structure, that is

$$\begin{aligned} a[0]*y[n] = b[0]*x[n]&+b[1]*x[n-1]+\dots \\ +b[M]*x[n-M] &- a[1]*y[n-1]+\dots \\ &-a[N]*y[n-N] \end{aligned} \tag{S3}$$

where M and N are, respectively, the numerator and denominator degree and n is the sample number. Assuming $M = N$, the differential equations follow, with d state variables:

$$a[0]*y[n] = b[0]*x[n] + d[0][n-1] \tag{S4}$$

$$d[0][n] = b[1]*x[n] - a[1]*y[n] + d[1][n-1] \tag{S5}$$

$$d[1][n] = b[2]*x[n] - a[2]*y[n] + d[2[n-1] \tag{S6}$$

$$\vdots \tag{S7}$$

$$\begin{aligned} d[N-2][n] = b[N-1]*x[n]+ \\ -a[N-1]*y[n] + d[N-1][n-1] \end{aligned} \tag{S8}$$

$$d[N-1][n] = b[N]*x[n] - a[N]y[n] \tag{S9}$$

The transfer function is then expressed as:

$$Y(z) = \frac{b[0] + b[1]*z^{-1} + \dots + b[M]*z^{-M}}{a[0] + a[1]*z^{-1} + \dots + a[N]*z^{-N}} \tag{S10}$$

[1] Analog Filters Index. Online at `http://www.analog.com/en/index.html`

2. In the second phase an additional IIR filter [2] is applied to the data previously filtered by *lfilter*. The algorithm is shown below:

```
IIR_TC = 256b
back = data[0]
for i in range(1,ns):
    back = (
        back*(IIR_TC-1)+data[i])/IIR_TC
    data[i] = data[i]-back
return data
```

The variable *back* contains the background vector calculated at each iteration: for each channel and for each sample, the average is estimated using the next input value. This approach assumes that the entire signal stream is available, making it space inefficient. However, it can be easily modified to adapt the calculations to real-time processing, as occurs during the phase of use of the system by the end use. Figure S1 shows an example of the filtered signal obtained from the raw data of channel AF3 from subject 0 during the first run of session 1. The blue line indicates the initial signal 31 which, as underlined by Emotiv, includes a DC Offset around $4200\,\mu V$. With the application of the filters it is possible to obtain the signal with average amplitude equal to 0 (orange line) Berardinelli (2018).

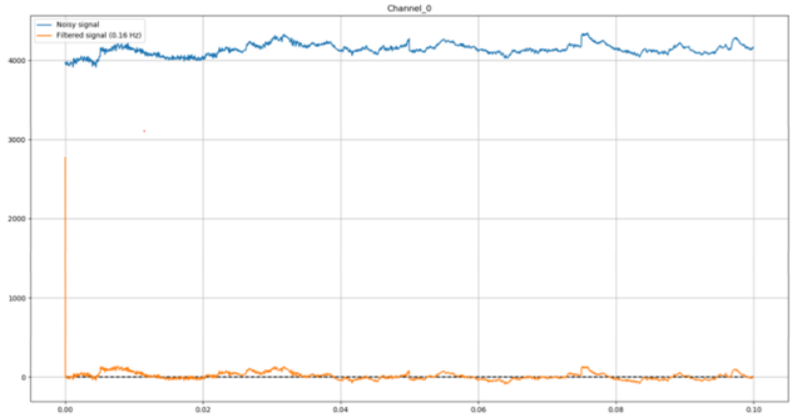

Figure S1: Example of raw signal (blue) after processing (orange).

2 FEATURE EXTRACTION

Feature extraction is been performed using all 14 electrodes supplied with the helmet. A band-pass filter is applied to each channel for splitting the signal into the following 5 frequency bands: σ (0 Hz– 0.14 Hz), θ (4 Hz–8 Hz), α (8 Hz–14 Hz), β (14 Hz–30 Hz), and γ (30 Hz–40 Hz). Thus, 70 distinct signals are obtained, 5 for each of the 14 channels of the helmet. A Butterworth band-pass filter is applied for the signal splitting:

```
scipy.signal.butter(N, [low, high], btype='band',
analog=False, output='ba')
```

[2] IIR Filters, ECE 2610 Signals and System. Online at this http://ece.uccs.edu/~mwickert/ece2610/lecture_notes/ece2610_chap8.pdf

The parameters have been tuned to obtain a band-pass filter (btype = 'band', 'low' and 'high' frequencies based on the selected band). The function calculates the numerator and denominator polynomials of the filter, respectively identified by b and a. Later the *lfilter* function was used, defined as:

```
scipy.signal.lfilter(b, a, x, axis=-1, zi=None)
```

which accepts the parameters b and a previously calculated and searches the data along a dimension with an IIR-type digital filter. Each of the 70 signals is broken down into time windows of 1 second with an overlap of 80%. Then, the *periodigram* function is applied to each window to calculate the power spectral density moving from the time to the frequency domain:

```
scipy.signal.periodogram(x,fs=128,
window='boxcar', nfft=None,
detrend='constant', return.onesided=True,
scaling='density', axis=-1)
```

where x represents the signal in the time domain and f_s is the sampling frequency relative to the sampled data. *Window* is the desired window to use, the default is 'boxcar'. *Nfft* is the length of the FFT used (if the value is 'none' the length of x will be used). *Detrend* specifies how to perform the detrend of each segment (the default value is 'constant'). *Return.onesided* if 'True' returns a one-sided spectrum for real data, if 'False' returns a two-sided spectrum. *Scaling* selects between the calculation of the power spectral density ('density') where P_{xx} has units of $\mathrm{V}^2\mathrm{Hz}^{-1}$ and the calculation of the power spectrum ('spectrum') where P_{xx} has units of V^2 (if x is measured in V and f_s is measured in Hz). By default it is 'density'. *Axis* determines the axis along which the periodigram is calculated. The function outputs two objects: the sampling frequency array and the power spectral density of x. The mathematical model underlying the periodigram function, for sufficiently small values of parameter T in the region $-1/2T < f < 1/2T$, could be represented by the following formula:

$$X_{1/T}(f) \triangleq \sum_{k=-\infty}^{\infty} X(f - k/T), \tag{S11}$$

which is precisely determined by the samples $x(nT)$ that span the non-zero duration of $x(t)$. For sufficiently large values of parameter N, $X_{1/T}(f)$ can be evaluated at an arbitrary close frequency by a summation of the form:

$$X_{1/T}\left(\frac{k}{NT}\right) = \sum_{k=-\infty}^{\infty} \underbrace{T \cdot X(nT)}_{\text{x(n)}} \cdot e^{-i2\pi \frac{kn}{N}}, \tag{S12}$$

where k is an integer. The periodicity of $e^{-i2\pi \frac{kn}{N}}$ allows this to be written very simply in terms of a Discrete Fourier Transform:

$$X_{1/T}\left(\frac{k}{NT}\right) = \underbrace{\sum_{n} x_N(n) \cdot e^{-i2\pi \frac{kn}{N}}}_{\text{DFT}}, \tag{S13}$$

sum over any n-sequence of lenght N, where x_N is a periodic summation: $x_N(n) \triangleq \sum_{m=-\infty}^{\infty} x(n - mN)$. When evalueted for all integers, k, between 0 and $N - 1$, the array:

$$S\left(\frac{k}{NT}\right) = \left|\sum_n x_N(n) \cdot e^{-i2\pi\frac{kn}{N}}\right|^2 \tag{S14}$$

is a *periodigram.*

3 MODELS OPTIMIZATION: INTERMEDIATE RESULTS

3.1 SVM model optimization results

Below, the grid of possible values for each parameter is reported:

- **SVM-linear**: ('kernel': ['linear']; 'C': [0.1, 1, 10]; 'tol': [1e-4, 1e-3, 1e-2]; 'shrinking': [True, False])
- **SVM-poly**: ('kernel': ['poly']; 'C': [0.1, 1, 10]; 'degree': [2,3,4]; 'gamma': ['scale', 'auto']; 'tol': [1e-4, 1e-3, 1e-2];'shrinking': [True, False])
- **SVM-rbf**: ('kernel': ['rbf']; 'C': [0.1, 1, 10]; 'gamma': ['scale', 'auto']; 'tol': [1e-4, 1e-3, 1e-2]; 'shrinking': [True, False])
- **SVM-linearsvc**: ('kernel': ['poly']; 'penalty': ['l1','l2']; 'loss': ['hinge', 'squared_hinge']; 'tol': [1e-4, 1e-3, 1e-2]; 'max_iter': [1000, 2000])

Below the results of the optimization procedure for all the SVC models.

percentile	accuracy	avg_precision	avg_recall	avg_f1_score	prediction time (s)
**5**	**0.92**	**0.93**	**0.93**	**0.92**	**2.0e-05**
10	0.92	0.92	0.92	0.92	2.0e-05
15	0.90	0.91	0.90	0.90	2.2e-05
20	0.90	0.91	0.90	0.90	2.6e-05
25	0.89	0.90	0.89	0.89	2.1e-05
30	0.87	0.89	0.87	0.89	3.9e-05
35	0.89	0.90	0.89	0.89	2.9e-05
40	0.89	0.90	0.89	0.88	3.4e-05
45	0.89	0.90	0.89	0.88	2.8e-05
50	0.88	0.90	0.88	0.88	2.9e-05
55	0.89	0.90	0.89	0.88	2.6e-05
60	0.88	0.89	0.88	0.88	2.0e-05
65	0.88	0.90	0.88	0.88	1.7e-05
70	0.88	0.90	0.88	0.88	1.9e-05
75	0.87	0.89	0.87	0.87	3.8e-05
80	0.87	0.88	0.87	0.87	2.0e-05
85	0.87	0.88	0.87	0.86	2.4e-05
90	0.85	0.87	0.85	0.85	1.9e-05
95	0.86	0.87	0.86	0.85	3.5e-05
100	0.86	0.88	0.86	0.86	2.9e-05

Table S1. Cross-validation results of the evaluation metrics for each subset of best parameters for SVC linear model.

percentile	accuracy	avg_precision	avg_recall	avg_f1_score	pred_time(s)
**5**	**0.92**	**0.92**	**0.92**	**0.912**	**1.5e-05**
10	0.91	0.92	0.91	0.91	1.9e-05
15	0.91	0.91	0.91	0.90	2.2e-05
20	0.87	0.89	0.87	0.87	6.0e-05
25	0.86	0.87	0.86	0.86	5.0e-05
30	0.87	0.89	0.87	0.87	5.6e-05
35	0.89	0.90	0.89	0.89	3.7e-05
40	0.89	0.90	0.89	0.88	4.1e-05
45	0.88	0.90	0.88	0.88	3.4e-05
50	0.89	0.90	0.89	0.89	4.6e-05
55	0.89	0.90	0.89	0.88	1.6e-05
60	0.88	0.90	0.88	0.88	1.9e-05
65	0.88	0.89	0.88	0.88	3.7e-05
70	0.88	0.90	0.88	0.88	1.9e-05
75	0.88	0.89	0.88	0.87	2.4e-05
80	0.88	0.89	0.88	0.87	4.6e-05
85	0.87	0.89	0.87	0.87	5.7e-05
90	0.87	0.88	0.87	0.87	5.1e-05
95	0.87	0.88	0.87	0.87	6.0e-05
100	0.87	0.88	0.87	0.87	6.5e-05

Table S2. Cross-validation results of the evaluation metrics for each subset of best parameters for SVC polynomial model.

percentile	accuracy	avg_precision	avg_recall	avg_f1_score	pred_time (s)
**5**	**0.92**	**0.92**	**0.92**	**0.92**	**4.3e-05**
10	0.91	0.92	0.91	0.91	4.7e-05
15	0.90	0.90	0.90	0.90	5.8e-05
20	0.89	0.89	0.89	0.89	6.7e-05
25	0.87	0.89	0.87	0.87	6.1e-05
30	0.90	0.90	0.90	0.89	2.0e-04
35	0.89	0.91	0.89	0.89	4.7e-05
40	0.89	0.90	0.89	0.89	1.1e-04
45	0.89	0.90	0.89	0.89	9.5e-05
50	0.89	0.90	0.89	0.89	1.1e-04
55	0.89	0.90	0.89	0.89	1.1e-04
60	0.88	0.89	0.88	0.88	1.3e-04
65	0.88	0.90	0.88	0.88	5.0e-05
70	0.88	0.89	0.88	0.88	6.0e-05
75	0.87	0.89	0.87	0.87	1.5e-04
80	0.89	0.90	0.89	0.89	2.9e-04
85	0.87	0.88	0.87	0.86	7.5e-05
90	0.89	0.89	0.89	0.89	3.9e-04
95	0.88	0.89	0.88	0.88	4.5e-04
100	0.86	0.88	0.86	0.86	8.1e-05

Table S3. Cross-validation results of the evaluation metrics for each subset of best parameters for SVC RBF model.

3.2 Decision Tree

Below, the grid of possible values for each parameter is reported:

percentile	accuracy	avg_precision	avg_recall	avg_f1_score	pred_time(s)
**5**	**0.91**	**0.92**	**0.91**	**0.91**	**1.3e-07**
10	0.90	0.91	0.90	0.90	4.7e-08
15	0.89	0.90	0.89	0.89	1.4e-07
20	0.89	0.90	0.89	0.89	1.2e-07
25	0.88	0.89	0.88	0.88	2.0e-07
30	0.88	0.89	0.88	0.87	1.4e-07
35	0.88	0.90	0.88	0.88	1.9e-07
40	0.87	0.89	0.87	0.87	1.5e-07
45	0.87	0.89	0.87	0.87	1.4e-07
50	0.87	0.89	0.87	0.86	1.7e-07
55	0.87	0.89	0.87	0.87	4.1e-06
60	0.86	0.88	0.86	0.86	1.8e-07
65	0.86	0.88	0.86	0.85	1.7e-07
70	0.86	0.88	0.86	0.86	2.0e-07
75	0.85	0.87	0.85	0.84	1.8e-07
80	0.85	0.87	0.85	0.84	2.4e-07
85	0.84	0.86	0.84	0.84	2.0e-07
90	0.83	0.85	0.83	0.82	2.1e-07
95	0.83	0.85	0.83	0.83	2.3e-07
100	0.83	0.86	0.83	0.83	2.2e-07

Table S4. Cross-validation results of the evaluation metrics for each subset of best parameters for SVC linearsvc model.

- 'criterion': ['entropy']; 'splitter': ['best', 'random']; 'max_depth': [3, 6, 9, 12, 15, None]; 'min_samples_split': [2, 3, 4, 5]; 'min_samples_leaf': [1, 2, 3, 4]; 'max_features': ['auto', 'sqrt', 'log2', None]; 'ccp_alpha': [0.0, 0.010, 0.020, 0.030]

Below the results of the optimization procedure.

percentile	accuracy	avg_precision	avg_recall	avg_f1_score	pred_time (s)
**5**	**0.78**	**0.81**	**0.78**	**0.76**	**9.0e-08**
10	0.75	0.80	0.75	0.73	4.7e-08
15	0.84	0.86	0.84	0.83	7.2e-08
20	0.80	0.82	0.80	0.79	6.9e-08
25	0.81	0.83	0.81	0.80	8.8e-08
30	0.79	0.82	0.79	0.79	1.2e-07
35	0.74	0.77	0.74	0.72	1.2e-07
40	0.73	0.75	0.73	0.72	9.7e-08
45	0.71	0.75	0.71	0.69	1.4e-07
50	0.75	0.78	0.75	0.74	1.1e-07
55	0.70	0.74	0.70	0.68	9.4e-08
60	0.66	0.70	0.66	0.63	1.5e-07
65	0.72	0.75	0.72	0.70	1.8e-07
70	0.73	0.76	0.73	0.71	1.5e-07
75	0.70	0.73	0.70	0.68	1.2e-07
80	0.77	0.78	0.77	0.76	1.5e-07
85	0.70	0.72	0.70	0.68	2.0e-07
90	0.72	0.75	0.72	0.70	2.1e-07
95	0.70	0.73	0.70	0.68	2.6e-07
100	0.72	0.75	0.72	0.70	3.0e-07

Table S5. Cross-validation results of the evaluation metrics for each subset of best parameters for DT model.

3.2.1 Random Forest

Below, the grid of possible values for each parameter is reported:

- 'criterion': ['entropy']; 'max_depth': [3, None]; 'min_samples_split': [2]; 'min_samples_leaf': [1]; 'n_estimators': [50, 100, 200]; 'bootstrap': [True, False]; 'oob_score': [True, False]; 'warm_start': [True, False]; 'ccp_alpha': [0.0]

Below the results of the optimization procedure.

percentile	accuracy	avg_precision	avg_recall	avg_f1_score	pred_time (s)
**5**	**0.88**	**0.90**	**0.88**	**0.88**	**3.8e-06**
10	0.88	0.90	0.88	0.87	7.2e-06
15	0.88	0.90	0.88	0.88	1.6e-05
20	0.86	0.88	0.86	0.85	1.5e-05
25	0.85	0.88	0.85	0.84	1.5e-05
30	0.84	0.88	0.84	0.83	1.4e-05
35	0.87	0.89	0.87	0.86	3.7e-06
40	0.88	0.90	0.88	0.87	1.4e-05
45	0.87	0.89	0.87	0.87	1.4e-05
50	0.88	0.90	0.88	0.87	1.4e-05
55	0.87	0.89	0.87	0.86	1.4e-05
60	0.86	0.89	0.86	0.86	1.4e-05
65	0.87	0.89	0.87	0.86	3.8e-06
70	0.86	0.89	0.86	0.86	1.5e-05
75	0.87	0.89	0.87	0.87	7.4e-06
80	0.87	0.89	0.87	0.86	1.4e-05
85	0.87	0.89	0.87	0.86	1.4e-05
90	0.86	0.88	0.86	0.86	7.3e-06
95	0.86	0.88	0.86	0.85	7.3e-06
100	0.85	0.88	0.85	0.85	1.4e-05

Table S6. Cross-validation results of the evaluation metrics for each subset of best parameters for RF model.

3.2.2 KNN

Below, the grid of possible values for each parameter is reported:

- 'n_neighbors': [5, 10, 20]; 'weights': ['uniform', 'distance']; 'algorithm': ['auto', 'ball_tree', 'kd_tree', 'brute']; 'leaf_size': [30, 60, 120]; 'p': [1,2,3].

Below the results of the optimization procedure.

3.2.3 MLP

For the MLP model, as mentioned above, a random search algorithm has been choosen: *Hyperband* from Keras Python library. The parameter ranges selected are shown below:

- 'n_layers': [1, 2, 4, 5]; 'n_dense_values': ['min'=32, 'max'=1024, 'step'=8]; 'drop_out_values': ['min'=0, 'max'=0.9, 'step'=0.1]; 'optimizer': ['adam', 'adamax']; 'output_activation': ['softmax', 'sigmoid'].

Below the results of the optimization procedure.

Finally, Table S9 shows the results of the 10-fold cross-validation processes performed on each classifier.

**percentile**	**accuracy**	**avg_precision**	**avg_recall**	**avg_f1_score**	**pred_time (s)**
5	0.88	0.90	0.88	0.88	4.9e-05
10	0.89	0.90	0.89	0.89	1.3e-04
15	0.89	0.90	0.89	0.89	1.4e-04
20	0.86	0.87	0.86	0.86	1.5e-04
25	0.87	0.88	0.87	0.87	1.5e-04
30	0.88	0.88	0.88	0.87	1.3e-04
35	0.89	0.89	0.89	0.89	1.6e-04
40	0.89	0.90	0.89	0.89	1.6e-04
45	0.89	0.90	0.89	0.89	1.7e-04
**50**	**0.90**	**0.91**	**0.90**	**0.90**	**1.7e-04**
55	0.90	0.91	0.90	0.90	1.5e-04
60	0.89	0.90	0.89	0.89	1.8e-04
65	0.89	0.90	0.89	0.89	1.7e-04
70	0.89	0.90	0.89	0.89	1.8e-04
75	0.89	0.90	0.89	0.89	1.9e-04
80	0.88	0.89	0.88	0.88	1.8e-04
85	0.87	0.88	0.87	0.87	2.0e-04
90	0.85	0.86	0.85	0.85	2.0e-04
95	0.83	0.85	0.83	0.83	2.1e-04
100	0.82	0.84	0.82	0.81	2.0e-04

Table S7. ross-validation results of the evaluation metrics for each subset of best parameters for KNN model.

**percentile**	**accuracy**	**avg_precision**	**avg_recall**	**avg_f1_score**	**pred_time (s)**
**5**	**0.91**	**0.91**	**0.91**	**0.91**	**2.9e-05**
10	0.89	0.91	0.89	0.88	2.6e-05
15	0.88	0.90	0.88	0.88	3.9e-05
20	0.86	0.88	0.86	0.86	3.6e-05
25	0.86	0.88	0.86	0.85	3.7e-05
30	0.90	0.91	0.90	0.90	4.1e-05
35	0.90	0.91	0.90	0.90	2.9e-05
40	0.89	0.91	0.89	0.88	3.5e-05
45	0.89	0.91	0.89	0.89	3.1e-05
50	0.91	0.91	0.91	0.90	3.1e-05
55	0.90	0.91	0.90	0.90	3.5e-05
60	0.89	0.90	0.89	0.89	3.2e-05
65	0.89	0.90	0.89	0.89	4.9e-05
70	0.90	0.91	0.90	0.90	3.1e-05
75	0.89	0.90	0.89	0.89	3.2e-05
80	0.89	0.90	0.89	0.89	4.2e-05
85	0.88	0.89	0.88	0.88	3.2e-05
90	0.88	0.89	0.88	0.88	4.0e-05
95	0.89	0.89	0.89	0.88	4.2e-05
100	0.88	0.89	0.88	0.88	3.8e-05

Table S8. Cross-validation results of the evaluation metrics for each subset of best parameters for MLP model.

Fig.S2 (left side) shows the 8 curves relating to the average accuracy recorded by the best models as the percentile of selected features varies. As the percentile of features increases it's possible to see a general trend characterized by a significant decrease in performance. All the proposed models, except of the KNN model, obtain the best performance on datasets composed of a value ranging from 5% to 15% of the best features. Specifically, the svm_linear model increases accuracy by 7% on a dataset consisting of 5% of the

Table S9. Results for best percentile score for each classifier.

	percentile	accuracy	avg_precision	avg_recall	avg_f1_score	pred_time (s)
SVM linear	**5**	**0.92**	**0.93**	**0.93**	**0.92**	**2.0e-05**
	15	0.90	0.91	0.90	0.90	2.2e-05
	25	0.89	0.90	0.89	0.89	2.1e-05
	50	0.88	0.90	0.88	0.88	2.9e-05
	75	0.87	0.89	0.87	0.87	3.8e-05
	100	0.86	0.88	0.86	0.86	2.9e-05
SVM poly	**5**	**0.92**	**0.92**	**0.92**	**0.912**	**1.5e-05**
	15	0.91	0.91	0.91	0.90	2.2e-05
	25	0.86	0.87	0.86	0.86	5.0e-05
	50	0.89	0.90	0.89	0.89	4.6e-05
	75	0.88	0.89	0.88	0.87	2.4e-05
	100	0.87	0.88	0.87	0.87	6.5e-05
SVM rbf	**5**	**0.92**	**0.92**	**0.92**	**0.92**	**4.3e-0**
	15	0.90	0.90	0.90	0.90	5.8e-05
	25	0.87	0.89	0.87	0.87	6.1e-05
	50	0.89	0.90	0.89	0.89	1.1e-04
	75	0.87	0.89	0.87	0.87	1.5e-04
	100	0.86	0.88	0.86	0.86	8.1e-05
SVM linearsvc	**5**	**0.91**	**0.92**	**0.91**	**0.91**	**1.3e-07**
	15	0.89	0.90	0.89	0.89	1.4e-07
	25	0.88	0.89	0.88	0.88	2.0e-07
	50	0.87	0.89	0.87	0.86	1.7e-07
	75	0.85	0.87	0.85	0.84	1.8e-07
	100	0.83	0.86	0.83	0.83	2.2e-07
DT	5	0.78	0.81	0.78	0.76	9.0e-08
	**15**	**0.84**	**0.86**	**0.84**	**0.83**	**7.2e-08**
	25	0.81	0.83	0.81	0.80	8.8e-08
	50	0.75	0.78	0.75	0.74	1.1e-07
	75	0.70	0.73	0.70	0.68	1.2e-07
	100	0.72	0.75	0.72	0.7	3.0e-07
RF	**5**	**0.88**	**0.90**	**0.88**	**0.88**	**3.8e-06**
	15	0.88	0.90	0.88	0.88	1.6e-05
	25	0.85	0.88	0.85	0.84	1.5e-05
	50	0.88	0.90	0.88	0.87	1.4e-05
	75	0.87	0.89	0.87	0.87	7.4e-06
	100	0.85	0.88	0.85	0.85	1.4e-05
KNN	5	0.88	0.90	0.88	0.88	4.9e-05
	15	0.89	0.90	0.89	0.89	1.4e-04
	25	0.87	0.88	0.87	0.87	1.5e-04
	**50**	**0.9**	**0.91**	**0.90**	**0.90**	**1.7e-04**
	75	0.89	0.90	0.89	0.89	1.9e-04
	100	0.82	0.84	0.82	0.81	2.0e-04
MLP	**5**	**0.91**	**0.91**	**0.91**	**0.91**	**2.9e-05**
	15	0.88	0.90	0.88	0.88	3.9e-05
	25	0.86	0.88	0.86	0.85	3.7e-05
	50	0.91	0.91	0.91	0.90	3.1e-05
	75	0.89	0.90	0.89	0.89	3.2e-05
	100	0.88	0.89	0.88	0.88	3.8e-05

best features, svm_poly records an increase of 5.2% on the same subset of features, similarly svm_rbf and svm_sigmoid records an improvement of 6.6% and 9.3% respectively. DT model appears to be the most sensitive to feature selection exploiting an improvement in accuracy equal to 16.6% on the subset relating

to the 15% of the best features. RF, MLP and KNN respectively achieve an increase in accuracy of 3.4%, 3.5% and 10%, with the KNN model differing from the others for an optimal subset of feature equal to the best 50%. Fig.S2 (right side) show the 8 curves relating to the difference, in terms of delta precision, recorded by the best models as the percentile of selected features varies. Delta precision is calculated as the difference between the average precision on the two classes.

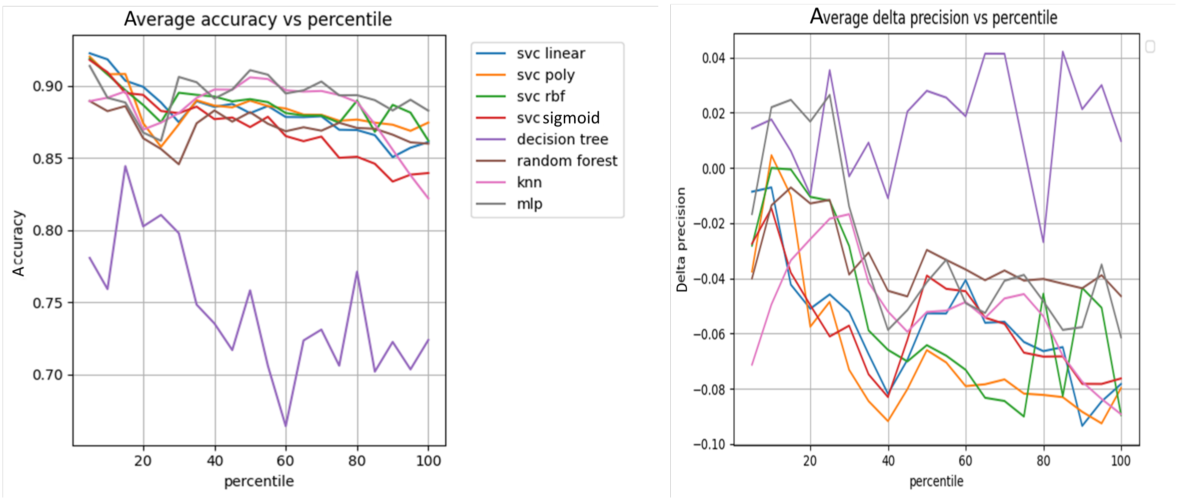

Figure S2: Average accuracy vs percentile (left side) and delta precision vs percentile (right side) recorded by best models extracted.

Although the accuracy metric is the main evaluation metric to determine the quality of a classifier, the precision achieved on each class is a fundamental parameter to ensure that the model is suitable for practical applications. Generally, except in particular contexts, the goal to be achieved ideally consists in obtaining a symmetrical classifier as regards the precision with which the different classes of data are predicted. The graph shows a general trend characterized by a growing imbalance in favor of the positive interaction class as the percentile of features considered increases, with the only exception of the DT model which appears to be independent of this dynamic. The svm_linear model turns out to be the most balanced in its optimized state, recording a delta of under one percentage point, confirming itself as the best model in terms of classification quality. Anyway, all the models analyzed, in their optimized configuration, do not deviate by more than 5% from the equilibrium point. The drift of the models towards an increase in the precision delta is probably due to the noise contained in the data. It is usually directly proportional to the number of inessential features and so the emotional state referring to the interaction with the negative robot is more likely affected because it's characterized by a lower amplitude of the quantities involved (less brain activity). Therefore, in a context where maximum accuracy is required, without limitations in terms of computational resources, the two models svm_linear and svm_poly are the best. In particular, the svm_linear model, in addition to being the best in terms of accuracy (92.23%), appears to be the most balanced in terms of predictive precision between the two data classes ($delta < 1\%$). In a context where high scalability is required, with limited computational resources, the svm_sigmoid model is undoubtedly the most cheap model characterized by the best accuracy/time ratio, it deviates by just under 0.5% from the best model in terms of accuracy but using between 100 and 200 times less time. As already fixed by the state of the art and by the researches carried out in the field of BCI, the models based on SVM appear to be the best candidates for solving machine learning problems based on the recognition of the human

emotional spectrum through EEG signals. It can also be observed that the models based on DT, given the strong susceptibility to noise contained in the data, are not very efficient in solving the proposed problem. Concluding, we may deduce from Fig.S2 that the DT model is unsuitable for the task, but we get similarly good results for the other optimized models with a statistically significant difference computed using the One-Way ANOVA test (The f-ratio value is 94.61 with a 2.07 f-critic). The significance level is $.00001$. The finding is statistically significant ($p < .05$).

REFERENCES

Berardinelli, M. (2018). Classificazione di segnali bci con training set selection e support vector machine

Butterworth, S. et al. (1930). On the theory of filter amplifiers. *Wireless Engineer* 7, 536–541
